# Beyond Delirium: Sporadic Creutzfeldt-Jakob Disease Revealed by Progressive Neurological Decline After Diabetic Ketoacidosis

**DOI:** 10.7759/cureus.113763

**Published:** 2026-08-01

**Authors:** FNU Abdul Rehman, Muhammad Zeeshan, Muhammad Usman Siddique, Kaleem Khaan

**Affiliations:** 1 Internal Medicine, Mayo Hospital, Lahore, PAK; 2 Internal Medicine, Continental Medical College, Lahore, PAK

**Keywords:** delirium, diabetes, diabetic ketoacidosis, myoclonus, rapidly progressive dementia

## Abstract

Creutzfeldt-Jakob disease (CJD) is a rare, rapidly progressive, fatal prion disorder that may initially mimic delirium, metabolic encephalopathy, psychiatric illness, or more common neurodegenerative conditions. Diagnostic recognition is particularly difficult when an acute medical illness provides an apparently plausible explanation for confusion. We report the case of a 74-year-old woman with insulin-dependent diabetes mellitus who was admitted after being found collapsed at home with vomiting and was diagnosed with diabetic ketoacidosis (DKA). Although the metabolic disturbance resolved, collateral history revealed several months of progressive dizziness, gait instability, recurrent falls, and visual hallucinations. During admission, she developed worsening cognitive decline, ataxia, myoclonus, reduced speech output, functional dependence, and eventual incontinence. Initial brain imaging was not diagnostic; however, neuroradiology review identified subtle basal ganglia and thalamic signal abnormalities on diffusion-weighted imaging (DWI) and fluid-attenuated inversion recovery (FLAIR) sequences. Cerebrospinal fluid (CSF) real-time quaking-induced conversion (RT-QuIC) testing returned positive, supporting the diagnosis of sporadic CJD. This case highlights the importance of reconsidering apparent delirium when neurological decline progresses despite correction of metabolic disturbance or exclusion of infection. Progressive cognitive impairment with hallucinations, ataxia, and myoclonus should prompt evaluation for rapidly progressive dementia, including sporadic CJD.

## Introduction

Creutzfeldt-Jakob disease (CJD) is a rare, rapidly progressive, fatal prion disorder that can present with cognitive decline, behavioral change, visual symptoms, ataxia, myoclonus, and eventual akinetic mutism. Early recognition is challenging because initial manifestations may be non-specific and can resemble delirium, psychiatric illness, metabolic encephalopathy, or more common neurodegenerative disorders [1]. This diagnostic difficulty is particularly relevant in older adults with acute medical illness, in whom metabolic or infectious triggers may provide an apparently sufficient explanation for confusion [2,3].

We report this case to highlight the diagnostic importance of persistent neurological deterioration after correction of an apparent metabolic precipitant. The objective is to emphasize that progressive cognitive decline with hallucinations, ataxia, and myoclonus should prompt early consideration of rapidly progressive dementia, including sporadic CJD, even when an initial diagnosis, such as diabetic ketoacidosis (DKA)-associated delirium, appears clinically plausible.

## Case presentation

A 74-year-old woman with insulin-dependent diabetes mellitus, chronic obstructive pulmonary disease, and a previous transient ischemic attack was admitted after being found collapsed at home with vomiting. Admission investigations confirmed DKA, with a blood glucose concentration of 24.7 mmol/L, blood ketones of 4.9 mmol/L, venous pH of 7.24, and bicarbonate of 14 mmol/L. The DKA resolved with standard medical management. Initial concern for urinary tract infection was not supported by subsequent evaluation; inflammatory markers improved, cultures were negative, and she had no fever or localizing infectious symptoms. During early admission, she was eating and drinking, had bowel movements, and mobilized with assistance.

Collateral history revealed a decline that had preceded hospitalization. Until earlier that year, she had lived independently and managed household responsibilities, finances, medications, and caregiving duties. After her husband’s death several months earlier, she initially remained functional, but later developed progressive dizziness, gait instability, and recurrent falls. Family members also noted new visual hallucinations involving her late husband, representing a clear departure from baseline. Despite these symptoms, she had continued to self-manage insulin therapy, as she had done for many years.

At initial neurological assessment, her Glasgow Coma Scale (GCS) score was 15/15. She could communicate, follow commands, and mobilize with assistance. There were no gaze abnormalities. Examination demonstrated gait ataxia and intermittent upper limb myoclonus. Limb power was 4/5 throughout, reflexes were preserved, and there were no focal neurological deficits. There was no neck stiffness or other evidence of meningeal irritation.

Although the metabolic disturbance improved, her neurological status continued to deteriorate. Over subsequent weeks, she developed worsening confusion, increasingly frequent visual hallucinations, progressive gait impairment, and episodes of reduced responsiveness with jerking movements. Functional decline became more marked, with loss of independent mobility, increasing dependence for activities of daily living, and eventual incontinence. Speech output diminished, and she became largely nonverbal. Further examination showed spontaneous upper limb myoclonus, more prominent on the left, and marked impairment in coordination. Although she could lift both upper limbs against gravity, she could not reliably perform finger-to-nose testing. Difficulty initiating eye opening and features suggestive of apraxia were also observed.

**Figure 1:**
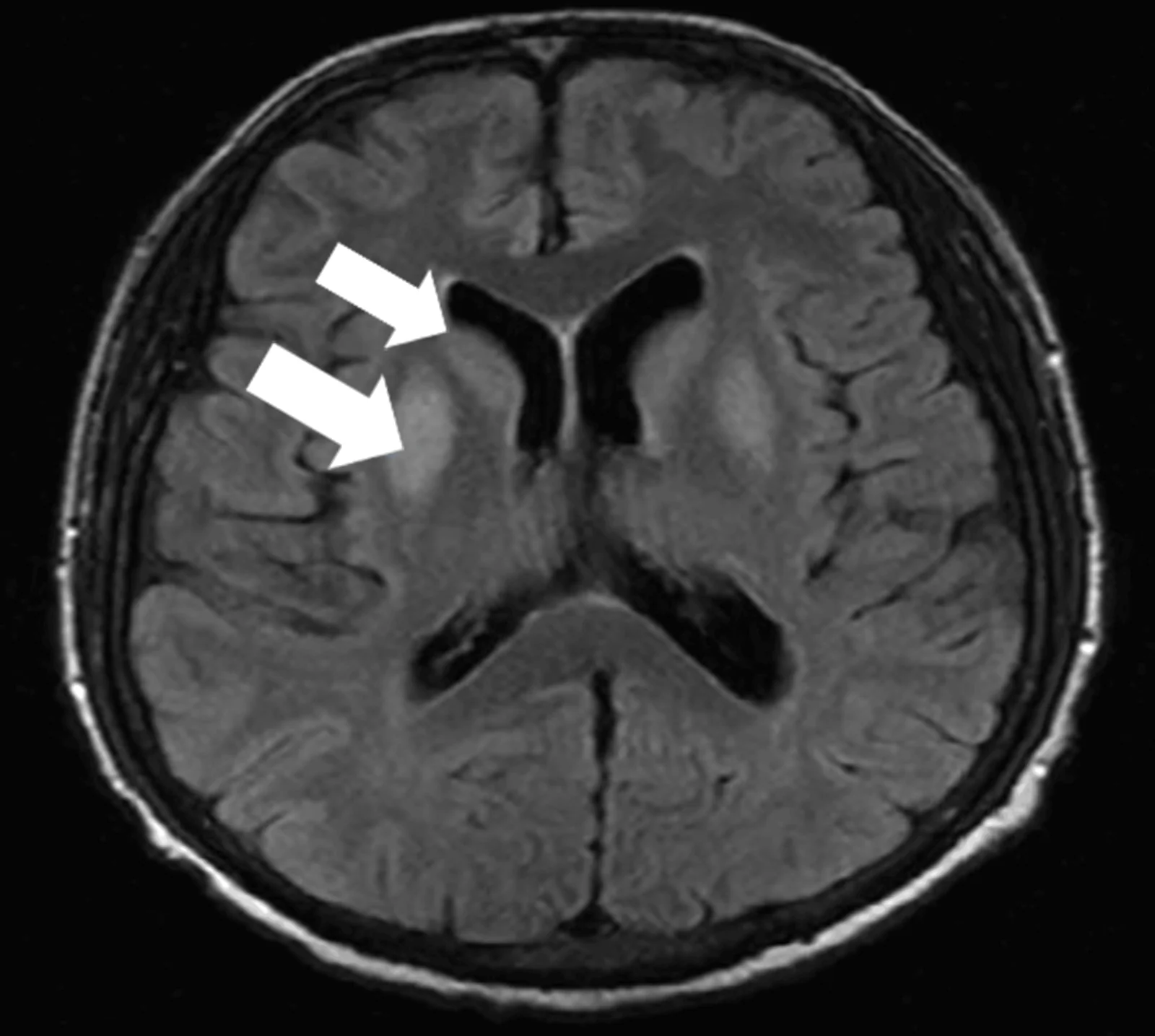
Axial FLAIR MRI brain showing bilateral basal ganglia hyperintensities, with white arrows highlighting representative signal abnormalities involving the caudate nuclei and putamina FLAIR, fluid-attenuated inversion recovery; MRI, magnetic resonance imaging

Initial investigations focused on potentially reversible causes of rapidly progressive cognitive decline. Computed tomography (CT) of the head was reported as showing no acute intracranial abnormality. Cerebrospinal fluid (CSF) analysis and infectious screening did not support an inflammatory, infectious, or reversible serological cause of encephalopathy. The key metabolic, CSF, and infectious investigations are summarized in Table 1. Magnetic resonance imaging (MRI) of the brain was initially reported as showing no acute abnormality, acute ischemia, significant chronic cerebrovascular disease, or mass lesion. However, given the combination of rapidly progressive cognitive decline, cerebellar dysfunction, visual hallucinations, and myoclonus, the imaging was reviewed by a neuroradiologist. Re-evaluation identified subtle signal abnormalities involving the basal ganglia, particularly the caudate nuclei, with additional involvement of the putamina and posteromedial thalami on diffusion-weighted imaging (DWI) and fluid-attenuated inversion recovery (FLAIR) sequences. Representative FLAIR abnormalities are shown in Figure 1. Contrast-enhanced MRI was reported as showing no meningeal or parenchymal enhancement. Electroencephalography (EEG) was performed; however, the report was not available before the patient’s death.

**Table 1:** Key metabolic, cerebrospinal fluid, and infectious investigations CSF, cerebrospinal fluid; HIV, human immunodeficiency virus

Investigation	Result	Reference range / Expected result
Blood glucose	24.7 mmol/L	3.9-7.8 mmol/L
Blood ketones	4.9 mmol/L	<0.6 mmol/L
Venous pH	7.24	7.32-7.43
Serum bicarbonate	14 mmol/L	22-29 mmol/L
CSF protein	0.34 g/L	0.15-0.45 g/L
CSF glucose	8.4 mmol/L	Approximately 60%-70% of serum glucose
CSF white blood cells	0 cells/µL	0-5 cells/µL
CSF red blood cells	147 cells/µL	0 cells/µL
CSF microbiology and virology	Negative	Negative
HIV serology	Negative	Negative
Syphilis serology	Negative	Negative

The clinical syndrome and imaging findings raised strong suspicion for sporadic CJD, and CSF real-time quaking-induced conversion (RT-QuIC) testing was requested. Management remained supportive and symptom-directed, with involvement of palliative care and speech and language therapy, as well as consideration of clonazepam for troublesome myoclonus. Following discussion with her son, consent was obtained for prion protein gene (PRNP) analysis, with blood samples retained for future testing and research.

Despite supportive management, her condition continued to deteriorate. She subsequently developed a low-grade fever, rising inflammatory markers, acute kidney injury, and prolonged unresponsiveness. In view of her advanced neurological decline and poor prognosis, goals-of-care discussions were held with her son, who held lasting power of attorney for healthcare decisions. A transition to comfort-focused care was agreed. CSF RT-QuIC testing subsequently returned positive, supporting the diagnosis of sporadic CJD. She died three days after initiation of end-of-life care.

## Discussion

CJD is a rare, uniformly fatal prion disease characterized by rapidly progressive neurodegeneration. Sporadic CJD accounts for most cases and commonly presents with rapidly progressive cognitive decline, myoclonus, cerebellar dysfunction, visual symptoms, pyramidal or extrapyramidal signs, and eventual akinetic mutism [3]. Its early features may be non-specific, making recognition particularly difficult in older adults with acute metabolic derangements or intercurrent infectious illness.

The main educational value of this case lies in the diagnostic challenge created by the initial presentation. DKA and suspected urinary tract infection offered plausible explanations for confusion and functional decline. However, the patient’s neurological deterioration continued despite correction of the metabolic disturbance and the absence of evidence for ongoing infection [3]. This progression was the key clinical turning point. Persistent or worsening cognitive impairment after resolution of an apparent delirium trigger should prompt reconsideration of the diagnosis and evaluation for rapidly progressive dementia [4].

Collateral history was central to recognizing that the illness had not begun with DKA. Months before admission, the patient had developed progressive dizziness, gait instability, recurrent falls, and visual hallucinations. These symptoms suggested an evolving neurological syndrome rather than acute metabolic encephalopathy alone [2,5]. The later emergence of myoclonus, worsening ataxia, speech impairment, apraxic features, incontinence, and near-mutism further supported a rapidly progressive neurodegenerative process. In this context, the combination of cognitive decline, cerebellar dysfunction, visual hallucinations, and myoclonus was highly suggestive of prion disease [1].

Neuroimaging was also instructive. The initial MRI study was not reported as diagnostic, but specialist re-review identified subtle abnormalities involving the caudate nuclei, putamen, and posteromedial thalamus on DWI and FLAIR sequences [6]. This highlights an important practical lesson: early CJD-related imaging abnormalities may be subtle and can be missed if clinical suspicion is low. When rapidly progressive cognitive and motor decline remains unexplained, MRI should be reviewed in direct correlation with the clinical syndrome.

CSF RT-QuIC testing provided strong diagnostic support in this case. Although EEG may be supportive, it is not always available within a clinically useful timeframe and may be non-specific, particularly early in the disease course. Diagnosis increasingly depends on integrating the clinical pattern, MRI findings, CSF analysis, and exclusion of more treatable mimics [1,7]. Although no disease-modifying treatment exists for sporadic CJD, timely diagnosis remains important because it enables appropriate counseling, symptom control, specialist support, and palliative planning.

A limitation of this report is that the original CT images and complete MRI dataset were unavailable for inclusion; however, a representative FLAIR image demonstrating the diagnostically relevant basal ganglia abnormalities is provided. This case emphasizes that delirium should remain a dynamic diagnosis rather than a fixed label. In older adults, metabolic or infectious triggers may coexist with an underlying neurological disorder. Progressive decline despite correction of these triggers, especially when accompanied by myoclonus, ataxia, visual hallucinations, and characteristic basal ganglia or thalamic MRI changes, should prompt early consideration of CJD and other causes of rapidly progressive dementia.

## Conclusions

This case highlights the importance of maintaining a broad differential diagnosis in older adults presenting with apparent delirium in the setting of acute metabolic illness. While diabetic ketoacidosis initially provided a plausible explanation for the patient's confusion, the persistence and progression of neurological symptoms despite correction of the metabolic disturbance prompted further evaluation and ultimately led to the diagnosis of sporadic Creutzfeldt-Jakob disease. Progressive cognitive decline accompanied by visual hallucinations, ataxia, and myoclonus should raise suspicion for rapidly progressive dementia, particularly when initial investigations are unrevealing. Careful collateral history, repeated neurological assessment, close clinicoradiological correlation, and timely use of RT-QuIC testing are essential for establishing the diagnosis. Although no disease-modifying treatment exists, early recognition facilitates appropriate counseling, symptom management, and palliative care planning for patients and their families.
